# Adaptation and validation of the Johnson-Lecci scale to assess anti-white bias among black UK minority group members

**DOI:** 10.1371/journal.pone.0277077

**Published:** 2022-11-28

**Authors:** Kim Dierckx, Alain Van Hiel, James D. Johnson, Len Lecci, Barbara Valcke, Eva Kefilwe Sekwena

**Affiliations:** 1 Department of Developmental, Personality and Social Psychology, Faculty of Psychology and Educational Sciences, Ghent University, Ghent, Belgium; 2 The Weber Group, Sellersburg, Indiana, United States of America; 3 Department of Psychology, University of North Carolina Wilmington, Wilmington, North Carolina, United States of America; 4 Department of Industrial and Organisational Psychology with Labour Relations Management, Northwest University, Potchefstroom, South Africa; Oslo University, NORWAY

## Abstract

The present study (total *N* = 901) set out to construct and validate a culturally sensitive instrument to examine anti-White bias among Black UK minority group members. Our novel measure of anti-White bias–which we called the AWB scale–was based upon the Johnson-Lecci scale (JLS; 2003) a questionnaire designed to measure anti-White attitudes among Black Americans. Studies 1 and 2 provided converging evidence for the AWB’s four-factor dimensionality, its structural characteristics, its temporal stability and its external validity in Black UK samples, attesting to the consistency of minorities’ experience of anti-majority bias in two very different societal contexts. Moreover, Study 3 evidenced our measure’s utility for understanding reactions to various relevant contemporary societal events. Theoretical contributions to the literature on intergroup bias are delineated and compared with majority-to-minority prejudice.

## Introduction

Negative intergroup attitudes and hostile intergroup behavior are two of the most extensively studied topics in social psychological literature [1–9]. Many research reports have focused on personality factors that may engender intergroup negativity [10–13]. This particular area of research has yielded a rich characterization of the traits, emotions and antecedents that feed the susceptibility to develop intergroup bias [14–18]. Yet, it should be noted that minority group participants are substantially underrepresented in the extant literature on biased attitudes (but see [19, 20], for noteworthy exceptions). To address this lacuna in literature, the critical goal of the present study was to develop a questionnaire that could be used to measure bias held by Black UK minority group members against the White majority population (anti-White bias hereafter). Additionally, we examined the value of anti-White bias as an explanatory variable for attitudes towards some key contemporary social issues (e.g., responses to maltreatment of ingroup members or stances towards actions being undertaken to combat structural racism). By doing so, we integrated two research traditions–i.e., research on intergroup bias and scholarly literature linking social attitudes to reactions to concurrent societal events [21–23]. Taken together, the present examination should contribute to social psychological literature by investigating the cross-cultural generalizability and specificity of bias. In addition, our work should contribute to psychological research in general, by providing much-needed insight into the psychology and experiences of the Black minority group in the UK.

### Prior work on intergroup bias among minority group members

While the majority perspective has received the bulk of scholarly attention, some progress has been made over the last decades with respect to the scientific knowledge about biases held by minority group members. A first line of research has established that interpersonal contact experiences can be a powerful determinant of negative (and positive) intergroup attitudes among disadvantaged group members [24–26]–although slightly less powerful than among privileged group members [27]. Secondly, the role of threat has been examined in the context of minorities as well. In line with integrated threat theory [28], it has been revealed that realistic and symbolic threats–stemming from group differences such as perceived status [29] and outgroup norms [30]–positively predict negative intergroup attitudes among minority group members.

Although the above findings are undoubtedly interesting in their own right and have advanced our understanding of intergroup negativity as such, many outstanding questions related to the biases held by disadvantaged groups remain. For example, only a few studies have attempted to characterize bias among minority group members and relate it to personality correlates [19, 31]. The lack of empirical attention for minority group member biases is further exacerbated by the fact that there are virtually no culture-specific measures of bias–with the exception of Johnson and Lecci’s questionnaire (JLS) [20]. This is problematic for at least two reasons. First, it is well-known that individuals from different cultures can have highly divergent construals of the self, and more importantly, of other individuals and other social groups [32]. Secondly, although prejudice measures exist that have been validated in majority groups, such as the subtle or blatant prejudice scales [33], using them to quantify minority member bias can be challenging, because the very nature of prejudicial attitudes themselves may vary across ethnic-cultural groups too. And indeed, the work of Brigham [34] showed that, compared to White participants, interracial attitudes among Black Americans were more complex and comprised multiple dimensions. In a similar vein, Johnson and Lecci [20, 35] demonstrated that, whereas White intergroup bias was predominantly outgroup-directed (i.e., negative beliefs about and emotions towards the outgroup), Black intergroup bias was both outgroup-driven and ingroup-directed (i.e., exaggerated meta-perceptions and strong emphasis on the outgroup’s intentions to discriminate against Black people). Thus, it seems that Black intergroup bias is a multidimensional concept, and one that is not straightforwardly studied using existing majority member intergroup bias scales [33].

Hence, in the present study, we sought to develop a culture-specific measure to quantify anti-White biases held by Black UK minority group members (which we based on the aforementioned JLS scale; see ‘The Present Studies’). One relevant issue is then: How can anti-White bias be characterized and what are its features? Prior work by Johnson and Lecci [20] suggests that anti-White bias consists of (at least) four related but distinct dimensions: Ingroup-directed stigmatization and discriminatory expectations, outgroup-directed negative beliefs, a negative stance towards intergroup relations, and the use of negative verbal expressions toward the outgroup. These authors further demonstrated in a series of studies the empirical distinctiveness of these dimensions and their relevance for various daily-life behaviors (e.g., discriminatory expectations predicted perceived racism by White people, etc.). Furthermore, a closer look at the relevant literature provides some secondary evidence for Johnson and Lecci’s typology. For example, the content of the first two factors (ingroup-directed stigmatization and discriminatory expectations, outgroup-directed negative beliefs) aligns with Brigham’s [34] observation that biases held by Black Americans are partly shaped by how negatively they perceive to be viewed by the outgroup (i.e., the White majority). Relatedly, reluctance to engage in interracial relations has been shown to be a critical feature of intergroup bias as well by various authors, both in majority [36] and minority samples [37]. Finally, Swim and colleagues [38] found that, when asked about past racist experiences, participants often report hearing comments made about their racial group–a finding which aligns with the existence of the negative verbal expressions dimension.

In sum, it appears that Johnson and Lecci’s model of anti-White bias provides a promising framework to ground our scale construction process. Therefore, in the present research, we thus operationalized anti-White bias as a mixture of (meta-)stereotypes, beliefs, evaluations, attitudes, and behaviors that express a disproportionate inclination against the White majority.

### Intergroup bias as predictor of reactions towards race-related societal events

It is often argued that the validity of any questionnaire is reflected in the extent to which it improves our understanding of real-life behavior [39–41]. Hence, in the current study we additionally explored the predictive utility of anti-White bias to explain (self-reported) reactions to race-related events. In their seminal paper, Johnson and Lecci [20] showed that the JLS significantly predicted if and to what extent ambiguous negative behavior enacted by White people was attributed to racial discrimination intentions. This finding is extremely important given that the increasing diversity in many countries may be linked to greater incidence of negative confrontations between minority groups and the national majority. Moreover, as the number of minority group members grows, perceptions of threat and, therefore, potential for conflict will also increase. An investigation into factors determining the interpretation of the intentions of majority group members thus seems warranted. Johnson et al. [35] also found that that the JLS reliably predicts other reactions relevant to race-related events, such as the willingness to confront perceived White racists and the acceptance of Black anti-White discrimination. Albeit undoubtedly informative about the role of biased attitudes in minority members, these findings nevertheless leave three intriguing questions unanswered. First, the passages administered by Johnson and colleagues [20, 35] were *hypothetical* scenarios. It therefore remains to be seen whether the registered responses align with reactions to actual events, or to descriptive passages that allude to contemporary, racially-charged events (e.g., a report of a White policeman shooting an unarmed Black man). Secondly, these initial validation studies were undertaken at the beginning of the 21^st^ century, which was characterized by a highly different “racial landscape” than nowadays (in terms of police violence, racial tensions, demands for racial equality, etc.). Hence, a next logical step would be to move beyond hypothetical laboratory race-related content and examine if a measure based upon the initial JLS can predict reactions to contemporary race-related “hot topics”, such as police shootings or the heated debates about the removal of infrastructure reminiscent of Western countries’ colonial past. Lastly, the above relationships were obtained in the American context, and it remains to be demonstrated whether anti-White bias relates to similar reactions to ‘typically’ European events with high relevance for the UK Black population.

Given our assumptions about the cross-cultural generalizability of the dimensionality and characteristics of biased attitudes, we did expect that our measure of anti-White bias would significantly predict reactions to such contemporary race-related European events. For example, if the original JLS predicted attributions to discrimination in passages describing hypothetical negative outcomes for Black people in the U.S., it stands to reason that similar attributions should be made by those high in anti-White bias when confronted with actual negative outcomes for Black people in the UK and Europe (e.g., the substantial employment gap between African migrants and White European majority members [42]). Moreover, we additionally anticipated that our measure of anti-White bias would also significantly predict reactions to topics that are relevant for Black UK citizens in particular, such as the recent debates about apologies and compensations for African nations that have been colonized by European countries, or the latest attempts by the Premier |League racist to combat online harassment and abuse aimed at Black soccer players in the UK.

## The present studies

In sum, we sought to construct and validate a culturally sensitive instrument to examine anti-White bias among Black UK minority group members. To this end, three studies were conducted. Our approach was a top-down strategy, such that we sought to adapt the JLS [20] measure to the UK context, rather than develop a completely novel instrument.

Specifically, in Study 1, we administered the JLS in a sample of UK Black people, and we compared the structural fit with that of a sample collected among American Black people. Our aim was to identify items (1) with good psychometric properties, and (2) which displayed superior measurement invariance across samples. Then, in Study 2, new items were developed to replace those which were less well suited in the UK context and/or displayed poor psychometric qualities, and to boost emerging factors which were underrepresented in the retained Study 1 items. Furthermore, to increase our confidence in our new measure–which we called the Anti-White Bias scale or AWB–and its construct validity, we examined the relationships with variables closely related to the concept. Moreover, by re-soliciting the Study 2 participants two months later, we sought to further validate the AWB scale by providing evidence on its temporal stability. Finally, in Study 3, reactions to race-related contemporary societal events (e.g., police shooting an unarmed Black man, reactions to allegations of an ingroup member committing a crime, and various other cases which were hypothesized to be relevant to AWB) were also measured, to investigate our scale’s predictive validity for (self-reported) real-life behavior.

The materials, data files, and data scripts of all studies can be accessed through our Open Science webpage (https://osf.io/gafyk/?view_only=3b87c8e129584ea9add2bd202343c5b2). All studies–with the exception of the explorative Study 1 –were formally preregistered on aspredicted.org, and anonymized versions of these preregistrations can be found on our Open Science webpage. Our study was approved by the Ethical Committee of our faculty. All measures, manipulations and exclusions are reported.

## Study 1

### Method

#### Participants

Despite ongoing debate on the issue [43], the idea that a sample size of 300 participants is the minimum requirement for initial scale validation is gaining support in the social sciences [44, 45]. A such, for the Black UK sample, we recruited 328 Black citizens Note that we initially intended to study anti-White bias among European Black people. However, due to Prolific participant availability issues (i.e., 80–90% of the collected samples was found to be living in the UK), we decided to focus on the UK participants instead. All main analyses for the full collected samples, as well as an overview of the countries of residence of the other participants, can be found on our Open Science webpage (section “Full sample analyses” in the Supplementary Online Materials) on Prolific [46], who were paid 1£ for their cooperation. This sample was collected in spring 2020. Our main inclusion criteria were that participants were fluent in English, that they and/or their ancestors originated from Sub Saharan Africa or the Caribbean, and that they were currently residing in the UK. We excluded *n* = 12 participants because they either correctly guessed the hypothesis of our study or because their answer to this question did not make any sense (e.g., “Yes” or “Estimate”), *n* = 2 because one Worker’s IP address appeared three times in our data, and *n* = 65 because they did not fulfill at least one of the above inclusion criteria. Furthermore, *n* = 5 failed our attention check (“Please select the third response for this question”), which resulted in a final sample of *N* = 244 (84 males; age: *M* = 27.8, *SD* = 8.60, range = 18–63).

The Black US sample was collected in the course of 2017–2018 through Amazon Mechanical Turk [47] in the context of another study (*N* = 210; 95 males; age: *M* = 33.4, *SD* = 10.24, range = 19–72). All participants passed our attention checks, and no further exclusions need to be reported. Written informed consent was obtained from all participants prior to participation. See Table 1 for an overview of further descriptive statistics for both samples.

**Table 1:** Descriptive statistics for black UK and US samples (Study 1).

	Black UK	Black US
Mean: Age (*SD*) ***	**27.8 (8.60)**	**33.4 (10.24)**
Number of males (%) **	**84 (34.4)**	**95 (45.2)**
Monthly income (median)	1000–1500€	*na*
Education: (% of sample)		*na*
No degree	1.0	
High school	31.1	
Bachelor’s degree	54.1	
Master’s degree	11.9	
Phd or equivalent	2.0	
Mean: Political ideology (*SD*)	3.54 (1.37)	*na*
Mean: JLS (*SD*) ***	**35.08 (17.56)**	**41.78 (17.86)**

*Notes*. *N*_UK Blacks_ = 244. *N*_American Blacks_ = 210. Political ideology was rated on a 7-point self-placement scale (1 = liberal, 7 = conservative); anti-White bias (JLS) was rated on 4-point (US sample) and 5-point (UK sample) Likert scales respectively, ranging from 1 = strongly disagree, to 4/5 = strongly agree. JLS mean scores are expressed in percent of maximum possible scores (i.e., the proportion of the difference between the theoretical minimum and theoretical maximum of the scales) to allow for comparison across samples. Significant differences between samples are printed in bold.

***: *p* < .001.

### Data analysis and results

#### Exploratory structural equation modeling (ESEM)

To investigate the psychometric properties of the JLS in the Black UK minority group, we fitted an Exploratory Structural Equation Model (ESEM) with the Psych package v2.0.7 [48] in R [49]. The ESEM technique [50] is a hybrid form of Exploratory (EFA) and Confirmatory Factor Analysis (CFA), incorporating advantages of both data-analytic strategies, and, more importantly, lacking some of their limitations [51]. Specifically, ESEM first runs an exploratory factor analysis and then uses the obtained factor loadings to estimate a prespecified measurement model [52]. ESEM is thus highly suited when researchers have a certain set of preexisting assumptions about the relationship between latent variables and their observed indicators, yet there remains a degree of uncertainty associated with the analysis [50].

Initially, we excluded three original JLS items which were not suited for the UK context based on item content (“I have referred to Whites as “crackers””, “I have referred to a White person as a “honkey”” and “I have called a White “redneck””). Exploratory analyses (see Supplementary Online Materials on our Open Science Webpage) revealed that a three-factor model would best suit the data. As such, in the first part of the ESEM procedure, an EFA with GeominQ rotation was conducted, which yielded a three-factor solution (explaining 50% of the variance in JLS item scores). A total of five items were eliminated because they did not contribute to a simple factor structure and failed to meet the minimum criteria of having a primary factor loading of .4 or above, and/or a cross-loading of .3 or below [53]. More importantly, retaining these items would likely have deteriorated the fit of our Step 2 model. The removed items belonged to the *outgroup-directed negative beliefs* facet scale (i.e., “I believe that Whites smell”, “I have suspected Whites of trying to destroy something created by Blacks”, “I believe that the success of a White person is due to their color” and “I consider myself to be racist toward Whites”) and the *negative views toward ingroup-outgroup relations* facet scales (i.e., “I have blamed Whites for my problems or for the problems of other Blacks”). A second EFA was then conducted, using only the remaining 12 items. This analysis once more yielded a three-factor solution, which explained 61% of the variance in JLS item scores.

In the second part of the procedure, we fitted a confirmatory SEM model using the factor loadings obtained in Step 1. This final three-factor model showed acceptable fit (*χ*^*2*^(33) = 94, *p* < .001, *χ2*/*df* = 2.84; CFI = 0.96, TLI = 0.93; RMSEA = 0.08, 90% CI = [0.066, 0.108], SRMR = 0.03; note that RMSEA is on the higher side of what constitutes an acceptable model fit). See Table 2 for an overview of the retained items and their corresponding factor loadings.

**Table 2:** Summary of ESEM results (standardized factor loadings and item content) for retained items of anti-White bias (Study 1).

Item	Factor I	Factor II	Factor III
1. I believe that most Whites would love to return to a time in which Blacks had no civil rights.	**.814**		
2. I believe that most Whites really do support the ideas and thoughts of racist political groups.	**.817**		
3. I believe that most Whites really believe that Blacks are genetically inferior.	**.844**		
4. I believe that most Whites would discriminate against Blacks if they could get away with it.	**.862**		
5. I believe that most of the negative actions of Whites toward Blacks are due to racist feelings.	**.552**	.102	
6. I believe that most Whites would harm Blacks if they could get away with it.	**.846**		
7. I believe that most Whites think that they are superior to Black.	**.855**		
8. I look negatively upon those involved in inter-racial relationships.		**1.368**	-.696
9. I have referred to mixed couples as “sell outs.”		**.527**	-.205
10. I have spoken negatively about Whites without concern as to their feelings.	.170	.337	**.593**
11. I have made racial comments.		.251	**.664**
12. I have insulted a White person.		.184	**.599**

*Notes*. Factor I = Ingroup-Directed Stigmatization and Discriminatory Expectations. Factor II = Negative Views towards Intergroup Relations. Factor III = Negative Verbal Expression towards the Outgroup. For clarity, loadings of < .100 are not reported.

#### Measurement invariance

We next assessed the equivalence of the 12 retained items across the Black UK and US samples. A common approach to test whether a construct has the same meaning across groups is to fit a sequence of four nested models, whereby each successive model makes more stringent assumptions about measurement invariance [54, 55]. The first model in the series tests for *configural* invariance, or invariance of the pattern of loadings–meaning that all items load on the same factors in both groups. The second model then assumes that these factor loadings are equal across groups–which is known as *weak* or *metric* invariance. Two final models test for *strong* or *scalar* (equality of loadings and intercepts) and *strict* invariance (equality of loadings, intercepts, and latent means).

In the process of determining which level of invariance had been achieved, goodness-of-fit statistics were calculated for each individual model and then used for comparison to the next model in the sequence. Given that reliance on chi-square based likelihood ratio tests can be tedious, because they tend to over-reject acceptable models at large sample sizes [56], researchers have suggested the use of other criteria. Specifically, Chen [57] has argued that changes in CFI, RMSEA and SRMR of ≥0.01, ≥0.015 and ≥0.030 respectively signal lack of invariance of a more restricted model, compared to a more parsimonious previous one. Thus, we assessed measurement invariance based on changes in these fit statistics, while simultaneously observing “global” fit of the more restricted model (i.e., the acceptability of other fit indices).

Table 3 displays goodness-of-fit-statistics for the four models, as well as model comparisons based on LRTs, and differences in CFI, RMSEA and SRMR.

**Table 3:** Measurement invariance: Multi-group CFA fit indices (Study 1).

Model	χ^2^(*df*)	CFI	TLI	RMSEA	SRMR	Δ χ^2^	*Δ*CFI	*Δ*RMSEA	*Δ*SRMR
I = Configural invariance	230(102)	0.958	0.946	0.074	0.050				
II = Metric invariance	236(111)	0.959	0.951	0.071	0.052	6.23^*ns*^	0.001	0.004	0.002
III = Scalar invariance	236(120)	0.962	0.958	0.065	0.052	0.00^*ns*^	0.003	0.005	0.000
IV = Strict invariance	236(123)	0.963	0.960	0.064	0.052	0.00^ns^	0.001	0.002	0.000

*Note*. *N* = 454. *ns* = non-significant.

It was revealed that the fourth model displayed the best fit (*χ*^*2*^(123) = 236, *p* < .001, *χ2*/*df* = 1.92; CFI = 0.96, TLI = 0.96; RMSEA = 0.06, 90% CI = [0.051, 0.076], SRMR = 0.05). This observation was further corroborated by our series of nested model tests investigating measurement invariance. As can be derived from Table 3, it was shown that goodness-of-fit did not deteriorate significantly when going from the first model in the series (which assumed weak or configural invariance or invariance of loadings) to the fourth model (which assumed strict invariance (or invariance of equality of loadings, intercepts, and latent means; all *Δχ*^*2*^s < 6.24, all *Δ*CFIs < .004, all *Δ*RMSEAs < 0.006, all *Δ*SRMRs < 0.003). Our results thus revealed that strict invariance was most likely for the three-factor solution with the 12 retained items. And, as such, they demonstrated that these items displayed similar psychometric properties across both societal contexts.

### Discussion

The main aim of Study 1 was to identify items pertaining to the original JLS [20] which could be incorporated in our new, UK measure of anti-White bias. The results highlighted 12 potential candidate items for our AWB scale, all of which displayed the preconceived psychometric properties. That is, they (1) loaded on theoretically anticipated latent factors, (2) they thereby contributed to an overall acceptable model fit, and (3) they were psychometrically equivalent across Black UK and American minority group member samples. Let it be noted that, for our purposes, weak invariance would have been sufficient, because our only inclusion criterion in this respect was that items should not load on different factors in both minority cultures–as this would violate the assumption that the basic “organization” of anti-majority bias would be similar in the two cultures. Nevertheless, results demonstrated that the group means for both minority groups on the latent factors which were obtained for the 12 retained items were comparable–thereby revealing scalar invariance (equivalence of factor loadings, intercepts, and means).

In sum, Study 1 thus identified a subset of potential candidate items to investigate anti-White bias. Yet, there were two more issues to be addressed. First, because the new measure of anti-White bias now only comprised 60% of the items of the original JLS, it was clear that the content coverage of the item pool needed to be re-expanded. Moreover, given that all items which originally loaded on the *negative beliefs* facet scale had been dropped due to poor structural properties, it was imperative that new items be generated to bolster this underrepresented facet. Again, it should be noted that we did not necessarily seek to preserve the factor structure obtained in Study 1, as this ran counter to our reasoning that the foundations of anti-White bias are supported in different cultures.

Study 2 thus served three purposes. First, we sought to develop a large pool of items which could be used to replace those removed in Study 1, and hence, further complete our AWB scale. Second, we intended to establish the validity of our final measure by investigating its relationships with variables closely related to the concept. We included four broad categories of constructs. A first set of variables referred to social categorization and social identification processes (i.e., Black racial identity, outgroup homogeneity, essentialism). Social categorization is typically seen as the cognitive determinant of stereotyping, discrimination and bias [58, 59]. In a similar vein, ingroup identification has also been related to increased outgroup prejudice [60, 61]. We thus expected that our questionnaire would be positively related to all these constructs. A second set of variables referred to intergroup contact and relations (i.e., quantity and quality of contact, intragroup and intergroup friendships, social distance). Given that positive interpersonal contact experiences are an effective strategy for reducing intergroup bias [62], we hypothesized that scores on our questionnaire would be negatively related to these constructs. A third set of variables referred to discrimination and unfairness perceptions (i.e., group relative deprivation, perceived group discrimination, procedural fairness perceptions). Group deprivation and disadvantage are indicative of group-directed societal bias, a feeling which is partially captured by the ingroup-directed stigmatization facet of the JLS [20]. Thus, we expected that scores on our questionnaire would be positively related to group relative deprivation and perceived group discrimination, and inversely related to procedural fairness perceptions enacted by societal actors towards Black people. Finally, to establish concurrent validity, we included two measures which have extensively been used to quantify prejudice among majority group members, i.e., the blatant prejudice and subtle prejudice scales [33]. Thirdly, and lastly, by re-soliciting the Study 2 participants two months later, we sought to examine the AWB’s temporal stability.

## Study 2

### Method

#### Participants

Study 2 was preregistered at https://aspredicted.org/blind.php?x=wj97ag. Sample size calculations and inclusion criteria were analogous to Study 1. Hence, we recruited *N* = 305 Black participants on Prolific (1£ renumeration). Written informed consent was obtained from all participants prior to participation. *N* = 3 participants were excluded because they either correctly guessed the hypothesis of our study or because their answer to this question did not make any sense, *n* = 20 because they did not fully complete the questionnaire, *n* = 1 because his/her IP address appeared three times in our data, and *n* = 35 because they did not fulfill at least one of our inclusion criteria, which resulted in a final sample of *N* = 246 Black UK citizens (86 males/157 females/3 non-binary; age: *M* = 29.0, *SD* = 8.72, range = 18–57). The majority of our participants had obtained a Bachelor’s (52.0%) or high school degree (24.8%; Master’s degree: 19.5%; no degree: 1.6%; PhD: 2.0). Furthermore, with respect to their political beliefs, participants rated themselves as 3.01 (*SD* = 1.27) on a 7-point, left-right self-placement scale (1 = left, 7 = right).

To further assess our final measure’s temporal stability, we contacted these 246 participants again about two months after the initial data collection and invited them to complete our new survey. Data collection was terminated three days after the third reminder had been sent out. Of the 246 contacted participants, an initial 208 (84.6%) participated in the follow-up part of our study (1£ remuneration). Fifteen participants were excluded because they did not fully complete the questionnaire, and another *n* = 3 were excluded because they completed the survey twice, which resulted in a final sample of *N* = 190 (77.2% of Study 1 T1 sample; 70 males/117 females/3 non-binary; age: *M* = 29.3, *SD* = 8.89, range = 18–57).

#### T1 measures

Besides the 12 items retained from Study 1 and our item pool which we created to replace the 8 removed items (see below), the following measures were also included in order to investigate their relationships with our anti-White measure. Unless stated otherwise, all items were scored on five-point Likert scales ranging from 1 = “Strongly disagree” to 5 = “Strongly agree”.

To measure **social categorization and identification processes**, we included a comprehensive measure of *Black racial identity*, namely, the Multidimensional Inventory of Black Identity (MIBI [63]). The MIBI reflects three central aspects of (African) identity, namely, centrality (a sample item is “In general, being Black is an important part of my self-image”), (private) regard (e.g., “I feel good about Black people”), and ideology–which further comprises the facet scales assimilationist philosophy (e.g., “Blacks who espouse separatism are as racist as White people who also espouse separatism” [reverse-scored]), humanist ideology (“Blacks and Whites have more commonalities than differences”), oppressed minority philosophy (“The same forces which have led to the oppression of Blacks have also led to the oppression of other groups”) and nationalist ideology (which refers to the extent to which participants stress the importance and uniqueness of being of African descent; e.g., “It is important for Black people to surround their children with Black art, music and literature”). We selected three items for each facet of Black racial identity. Furthermore, two items, adapted from Swart et al. [27], were implemented to quantify *perceived outgroup homogeneity*, i.e., the extent to which participants thought that White people were “all alike” (*M* = 1.80, *SD* = 0.91). Thirdly, to quantify the extent to which our participants exhibited *essentialist thinking*–which can broadly be defined as the conviction that the members of a racial/ethnic group all share the same underlying characteristics and are thus “all alike” [64]–we implemented 6 items adapted from the essentialist entativity scale developed by Roets and Van Hiel [65]. We particularly focused on the entativity facet of essentialism–i.e., beliefs about the uniformity and informational value of racial/ethnic group membership–because such convictions can reasonably be expected to relate significantly to JLS facets such as negative beliefs or ingroup-directed stigmatization. A sample item of this scale is “If you know to which racial group someone belongs, you know a lot about his/her personality” (*M* = 2.52, *SD* = 0.90).

To measure **intergroup contact and relations**, we implemented one item measuring the *quantity* of overall contact with White people (‘How often, if at all, do you have contact with White people?”; *M* = 4.35, *SD* = 0.83), three items measuring whether this contact was perceived as *positive* (e.g., “How often do you have friendly contact with White people?”; *M* = 4.02, *SD* = 0.86) and three items measuring whether this contact was perceived as *negative* (e.g., “How often do you have negative experiences with White people?”; *M* = 2.40, *SD* = 0.79). Two items assessed the prevalence and quality of *friendships with Black people*: “Please write down… The number of close Black friends in your friend circle?” (*M* = 5.18, *SD* = 12.01) and “How often do you spend time with these friends? [1 = never, 5 = very frequently]” (*M* = 3.30, *SD* = 1.15); and two items were implemented to measure the prevalence and quality of *friendships with White people*, i.e., “Please write down… The number of close White friends in your friend circle?” (*M* = 3.71, *SD* = 5.03) and “How often do you spend time with these friends?” (*M* = 3.08, *SD* = 1.17). Lastly, *social distance* was measured with six items, which we adapted from Hraba et al. [66]. A sample item is “I would be annoyed if my work colleagues were White people”. This scale’s reliability was high (*M* = 1.36, *SD* = 0.56).

Three scales were included to measure **discrimination and unfairness perceptions**. One item was adapted from Pettigrew & Meertens [33], to assess g*roup relative deprivation*, i.e., whether participants considered their ethnic group to be a disadvantaged group: “Would you say that over the last years people like yourself in your country have been… economically a lot better off, better off, the same, worse off, or a lot worse off… than most White majority members living there?” (reverse-scored; *M* = 2.59, *SD* = 0.86). *Perceived group discrimination* was measured with the nine-item Everyday Discrimination Scale [67], which was recently adapted by Dierckx et al. [68] to refer to perceptions of group discrimination. A sample item is “Black people are treated with less courtesy than other people are”. The reliability of this scale was high (*M* = 3.65, *SD* = 0.91). We included four items adapted from Liao and Rupp [69] measuring *procedural fairness*, i.e., the extent to which our participants perceived the societal institutions in their respective countries to act unbiasedly and rightfully towards Black people. A sample item is “I can count on the institutions in my country to have fair policies about issues that affect my ethnic group”. The reliability of this scale was high (*M* = 2.73, *SD* = 0.86).

Finally, we included two single-facet measures of **outgroup prejudice** among majority group members–i.e., the *blatant prejudice* and *subtle prejudice* scales [33]–and tailored them to our research population. Sample items are “White people always give preferential treatment to their own people” (blatant prejudice) and “I would not mind if a suitably qualified White person was appointed as my boss.” (subtle prejudice, reverse-scored). We included only those items which could be applied to minority group members’ attitudes towards the national majority group. For example, the item “Many other groups have come to [country] and overcome prejudice and worked their way up; [minority group] should do the same without special favor.”, pertaining to the original subtle prejudice scale of Pettigrew and Meertens [33], was dropped, because a history of immigration is inconsistent with the majority group status. As such, our final scales each comprised eight items. For an overview of the measures, we refer the reader to our Open Science webpage. The reliability of both scales was acceptable (blatant: *M* = 2.31, *SD* = 0.74; subtle: *M* = 2.25, *SD* = 0.61).

#### T2 measures

Participants completed the final 23-item AWB scale (see below, section ‘Scale Construction’; *M* = 2.94, *SD* = 0.76; *α* = .93). Furthermore, they also responded to the HEXACO-60 [70], a brief questionnaire designed to measure the HEXACO personality dimensions [71]. For all analyses involving personality, we refer the reader to the Supplementary Online Materials on our Open Science Webpage.

### Data analysis and results

#### Scale construction

Two types of items were generated: (1) items designed to replace the three items which were unsuited for the UK context (see Study 1), (2) and items designed to replace the five items which performed poorly in the Study 1 structural analyses. Our generation procedure consisted of three phases. First, we conducted a pilot study (*N* = 16 Black UK participants) in which participants were explained the context and main aims of our study. We then provided them with the removed items, and asked them to rewrite these items with special attention to applicability and interpretability, while keeping item content as close to the original as possible. Then, the main authors reviewed these items and added several more, based on the conceptual framework put forth by Johnson and Lecci [20]. In a final phase, we consulted with two experts in the field of social psychology–and outgroup bias specifically–as well as various researchers in our lab who were familiar with the scholarly literature on prejudice. They were asked to select those items which they found most suitable to replace the original ones. This approach resulted in the creation of a large pool of additional items (*n* = 18) which was then administered together with the 17 original JLS items. Our initial item pool is available in the Supplementary Materials on our Open Science webpage, together with the questionnaire and data of the pilot study.

Our ESEM procedure was identical to Study 1. First, an initial EFA (GeominQ rotation) was conducted, which yielded a four-factor solution (explaining 46% of the variance in JLS scores). A total of twelve items were deleted because they failed our inclusion criteria (having a primary factor loading of .4 or above, and/or a cross-loading of .3 or below). Then, to obtain the improved factor loadings for step 2, a second EFA on the remaining 23 items was performed. This analysis once more yielded a four-factor solution, which explained 53% of the variance in JLS scores. The obtained factor structure was easily interpretable because the factor labels proposed by Johnson and Lecci [20] suited three of the four factors, namely *ingroup-directed stigmatization* (sum of squared loadings [SS] = 3.66, % of explained variance = 16), *negative beliefs about the outgroup* (SS = 2.78, % explained variance = 12) and *negative verbal expressions towards the outgroup* (SS = 2.66, % explained variance = 12). To avoid any ambiguity in the naming of our factors, however, we hereafter label the first factor *perceptions of White anti-Black racism*, which–in our view–better fits the item content of this dimension. The last factor was also theoretically meaningful, and was labeled *negative attitudes/evaluations of the outgroup* (SS = 2.99, % explained variance = 13). Furthermore, the obtained pattern of factor loadings fit our initial inclusion criteria.

Secondly, we fitted a confirmatory SEM model using the obtained factor loadings. This final four-factor model showed acceptable fit (*χ*^*2*^(167) = 404, *p* < .001, *χ2*/*df* = 2.42; CFI = 0.94, TLI = 0.90; RMSEA = 0.07, 90% CI = [0.067, 0.085], SRMR = 0.04; note that RMSEA is on the higher side of what constitutes an acceptable model fit). See Table 4 for an overview of the retained items and their corresponding factor loadings.

**Table 4:** Summary of ESEM results (standardized factor loadings and item content) for final version of our AWB measure (Study 2).

Item	Factor I	Factor II	Factor III	Factor IV
1. I believe that most Whites would love to return to a time in which Blacks had no civil rights.	**.768**		.179	
2. I believe that most Whites really do support the ideas and thoughts of racist political groups.	**.804**		.141	
3. I believe that most Whites really believe that Blacks are genetically inferior.	**.713**	.159		
4. I believe that most Whites would discriminate against Blacks if they could get away with it.	**.770**	.212	-.108	
5. I believe that most of the negative actions of Whites toward Blacks are due to racist feelings.	**.465**	.324	-.155	
6. I believe that most Whites would harm Blacks if they could get away with it.	**.798**			
7. I believe that most Whites think that they are superior to Black.	**.739**	.203		
8. White people experience more success solely due to their “White privilege”.		**.779**		
9. I believe that Whites have had an advantage just because of their color.		**.837**		
10. Every White person is at an advantage simply because he/she is White.		**.791**		
11. Most of the mistreatment that Black people have faced is because of Whites.		**.498**	.107	.148
12. I have found myself thinking that the problems faced by Black people are simply because of Whites’ actions in the past.		**.553**		
13. I don’t like White people because of the bad things that they have done.		.106	**.685**	.199
14. I consider myself to hold a grudge towards Whites.			**.667**	.197
15. I believe that White people are all alike.			**.683**	
16. I’m not fond of Whites.			**.811**	.119
17. I consider myself to dislike Whites.			**.785**	.139
18. I have spoken negatively about Whites without concern as to their feelings.	.103		.162	**.509**
19. I have made racial comments.				**.583**
20. I have insulted a White person.			-.100	**.441**
21. I have expressed myself negatively about Whites on various occasions.			.142	**.777**
22. I have spoken negatively about Whites in general.			.135	**.748**
23. I have referred to White people by using negative terms.			.190	**.747**

*Notes*. Factor I = Perceptions of White Anti-Black Racism. Factor II = Negative Beliefs about the Outgroup. Factor III = Negative Attitudes Towards/Negative Evaluation of the Outgroup. Factor IV = Negative Verbal Expression towards the Outgroup. For clarity, loadings of < .100 are not reported.

For exploratory purposes, and in line with the debate we outlined in the Introduction about the multidimensionality of biases held by minority group members, we additionally compared the model fit of the obtained four-factor solution with a one-factor alternative. The results of this analysis revealed that the four-factor model significantly outperformed its one-dimensional counterpart (*χ*^*2*^ (6) = 1543, *p<* .001; fit of 1-factor model: *χ*^*2*^(230) = 2100, *p* < .001, *χ2*/*df* = 9.13; CFI = 0.50, TLI = 0.45; RMSEA = 0.18, 90% CI = [0.175, 0.189], SRMR = 0.18).

#### T1 external validity

We then proceeded to assess the relationships between participants’ scores on our AWB scale and relevant external criteria. Table 5 provides an overview of the correlations between our study’s variables. Correlations were also computed with and without covariates (age, gender, education, political beliefs), with virtually identical results. For the sake of parsimony, below we only report the results without background variables.

**Table 5:** Correlations among key variables in Study 2 (alpha coefficients on the diagonal).

	1	2	3	4	5	6	7	8	9	10	11	12	13	14	15	16.	17.	18.	19
1.AWB	*(*.*91)*																		
2.MIBI-C	**.21** ***	*(*.*89)*																	
3.MIBI-P	**.19** **	.61***	*(*.*62)*																
4.MIBI-A	**-.32** ***	.02	.06	*(*.*42)*															
5.MIBI-H	**-.46** ***	-.08	-.03	.45***	*(*.*56)*														
6.MIBI-O	**-.19** **	-.01	.07	.38***	.47***	*(*.*72)*													
7.MIBI-N	**.50** ***	.39***	.23***	-.25***	-.29***	-.11	*(*.*55)*												
8.Homogeneity	**.42** ***	.23***	.18**	-.20**	-.33***	-.01	.21***	*(*.*88)*											
9.Essentialism	**.33** ***	.08	.10	-.04	-.08	.09	.15*	.28***	*(*.*89)*										
10.Contact pos	**-.33** ***	-.09	-.06	.21***	.29***	.18**	-.15*	-.33***	-.12	*(*.*86)*									
11.Contact neg	**.31** ***	-.01	-.03	-.29***	-.23***	-.14*	.18**	.24***	.03	-.16*	*(*.*84)*								
12. Cont quant	**-.21** ***	-.08	-.04	.11	.17**	.13*	-.07	-.28***	-.13*	.59***	.09	*(n/a)*							
13.Wh.friends	-.13*	-.11	-.07	.19**	.21**	.18**	-.12	-.07	.03	.20**	-.03	.11	*(n/a)*						
14.SocDistance	**.47** ***	.12	.15*	-.26***	-.31***	-.13*	.27***	.36***	.16*	-.30***	.23***	-.26***	-.05	*(*.*81)*					
15.Rel. depr.	**.16** *	-.05	-.09	-.24***	-.12	-.11	.10	.03	-.07	.06	.19**	.10	.02	.02	*(n/a)*				
16.PD	**.66** ***	.29***	.18**	-.31***	-.43***	-.26***	.56***	.24***	.14*	-.23***	.30***	-.12	-.16*	.20**	.27***	*(*.*93)*			
17.PF	**-.42** ***	-.18**	-.10	.20**	.33***	.27***	-.37***	-.11	-.01	.12*	-.19**	.11	.13*	-.22***	-.22***	-.46***	*(*.*79)*		
18.Subtle Rac	**.57** ***	.24***	.20**	-.36***	-.39***	-.13*	.41***	.54***	.23***	-.45***	.25***	-.35***	-.15*	.42***	.12	.46***	-.29***	*(*.*72)*	
19.Blatant Rac	**.64** ***	.19**	.14*	-.24***	-.33***	-.09	.46***	.45***	.37***	-.33***	.27***	-.23***	-.07	.37***	.05	.52***	-.30***	.68***	*(*.*84)*

*Notes*. *N* = 246. Significant correlates of anti-White bias (23 items) are printed in bold. AWB = Anti-White bias. MIBI = Multidimensional Inventory of Black Identity. MIBI-C = centrality subscale. MIBI-P = private regard subscale. MIBI-A = assimilation subscale. MIBI-H = humanist ideology subscale. MIBI-O = oppressed minority philosophy subscale. MIBI-N = nationalist ideology. Homogeneity = perceived outgroup homogeneity. Contac pos = positive contact with White people. Contact neg = negative contact with White people. Cont quant = quantity of contact with White people. Wh.friends = number of close White friends. Soc distance = social distance. Rel. Depr. = relative group deprivation. PF = procedural fairness enactment towards Black people by societal actors. PD = perceived group discrimination. Subtle Rac = subtle racism. Blatant Rac = blatant racism.

*: *p* < .05.

**: *p* < .01.

***: *p* < .001.

With respect to our *social categorization and identification* variables, results revealed that AWB scores related strongly to African racial identity (*r*_centrality_ = .21, *p* < .001; *r*_private regard_ = .19, *p* = .003) and the various ideology facet scales (*r*_assimilation_ = -.32, *p* < .001; *r*_humanist_ = -.46, *p* < .001, *r*_oppressed minority_ = -.19, *p* = .003; *r*_nationalist_ = .50, *p* < .001). Furthermore, AWB was also strongly and positively related to perceived outgroup homogeneity (*r* = .42, *p* < .001) and essentialist entativity: (*r* = .33, *p* < .001).

With respect to *intergroup contact and relations*, it was shown that AWB was negatively associated with the number of close White friends (although not significantly; *r* = -.13, *p* = .050) and the time spent with them (*r* = -.24, *p* < .001). There were no significant associations between AWB and the number of close Black friends or the time spent with them (both *p*s > .350). Secondly, AWB also significantly predicted the quantity (*r* = -.21, *p* < .001) and quality of contact with White people (positive contact: *r* < -.33, *p* = .001; negative contact: *r* = .31, *p* < .001). Moreover, AWB was also strongly related to perceived social distance from White people (*r* = .47, *p* < .001).

With respect to *discrimination and unfairness perceptions*, we found that AWB was positively and significantly associated with perceived relative group deprivation (*r* = .15, *p* = .013) and perceived group discrimination (*r* = .66, *p* < .001), and negatively and significantly with political conservatism (*r* = -.18, *p* = .007) and perceived procedural fairness enactment towards Black people by societal actors (*r* = -.42, *p* < .001).

With respect to *outgroup prejudice*, we found that AWB was positively and significantly associated with subtle (*r* = .57, *p* < .001) and blatant racism (*r* = .64, *p* < .001).

#### T2 confirmatory factor analysis

We then assessed the structural properties of our anti-White bias scale at T2. To this end, we fitted a confirmatory SEM model using the same factor structure as identified above. This model fit the data acceptably (*χ*^*2*^(224) = 511, *p* < .001, *χ2*/*df* = 2.28; CFI = 0.90, TLI = 0.89; RMSEA = 0.08, 90% CI = [0.073, 0.092], SRMR = 0.07; note that RMSEA is on the higher side of what constitutes an acceptable model fit). See Table 6 for an overview of items and corresponding factor loadings.

**Table 6:** Summary of CFA results (standardized factor loadings and item content) for AWB (Study 2, T2).

Item	Factor I	Factor II	Factor III	Factor IV
1. I believe that most Whites would love to return to a time in which Blacks had no civil rights.	**.846**			
2. I believe that most Whites really do support the ideas and thoughts of racist political groups.	**.830**			
3. I believe that most Whites really believe that Blacks are genetically inferior.	**.840**			
4. I believe that most Whites would discriminate against Blacks if they could get away with it.	**.867**			
5. I believe that most of the negative actions of Whites toward Blacks are due to racist feelings.	**.654**			
6. I believe that most Whites would harm Blacks if they could get away with it.	**.787**			
7. I believe that most Whites think that they are superior to Black.	**.857**			
8. White people experience more success solely due to their “White privilege”.		**.811**		
9. I believe that Whites have had an advantage just because of their color.		**.806**		
10. Every White person is at an advantage simply because he/she is White.		**.780**		
11. Most of the mistreatment that Black people have faced is because of Whites.		**.579**		
12. I have found myself thinking that the problems faced by Black people are simply because of Whites’ actions in the past.		**.573**		
13. I don’t like White people because of the bad things that they have done.			**.846**	
14. I consider myself to hold a grudge towards Whites.			**.788**	
15. I believe that White people are all alike.			**.733**	
16. I’m not fond of Whites.			**.871**	
17. I consider myself to dislike Whites.			**.855**	
18. I have spoken negatively about Whites without concern as to their feelings.				**.735**
19. I have made racial comments.				**.613**
20. I have insulted a White person.				**.608**
21. I have expressed myself negatively about Whites on various occasions.				**.886**
22. I have spoken negatively about Whites in general.				**.841**
23. I have referred to White people by using negative terms.				**.736**

*Notes*. Factor I = Perceptions of White Anti-Black Racism. Factor II = Negative Beliefs about the Outgroup. Factor III = Negative Attitudes Towards/Negative Evaluation of the Outgroup. Factor IV = Negative Verbal Expression towards the Outgroup. For clarity, loadings of < .100 are not reported.

#### T2 measurement invariance

To assess the scale’s test-retest reliability, we tested for measurement invariance across measurement occasions. Obtaining a similar structure for our newly developed scale on different measurement occasions within the same group, would further testify to its usefulness to meaningfully quantify anti-White bias across time [55]. Hence, we ran the same procedure of nested model fitting as in Study 2, whereby a series of gradually more strict models (in terms of invariance assumptions) was evaluated and pitted against each other, based on the criteria proposed by Chen [57]–i.e., changes in CFI, RMSEA and SRMR of ≥0.01, ≥0.015 and ≥0.030. Table 7 displays goodness-of-fit-statistics for the four models, as well as model comparisons based on LRTs, and differences in CFI, RMSEA and SRMR.

**Table 7:** Measurement invariance: Multi-measurement CFA fit indices (Study 2, T2).

Model	χ^2^(*df*)	CFI	TLI	RMSEA	SRMR	Δ χ^2^	*Δ*CFI	*Δ*RMSEA	*Δ*SRMR
I = Configural invariance	1599(938)	0.900	0.889	0.061	0.066				
II = Metric invariance	1626(961)	0.899	0.891	0.060	0.073	26.96^*ns*^	0.001	0.001	0.007
III = Scalar invariance	1710(983)	0.890	0.884	0.062	0.078	81.17 ***	0.009	0.002	0.005
IV = Strict invariance	1710(980)	0.889	0.883	0.063	0.078	0.00 ^ns^	0.001	0.001	0.000

*Note*. *N* = 190.

*** = *p* < .001.

*ns* = non-significant.

As can be derived from this table, it was shown that, based on the proposed criteria, strict invariance could be assumed (*χ*^*2*^(980) = 1710, *p* < .001, *χ2*/*df* = 1.74; CFI = 0.90, TLI = 0.88; RMSEA = 0.06, 90% CI = [0.058, 0.068], SRMR = 0.08; comparison to the previous model in the sequence assuming strong invariance: (*Δχ*^*2*^(3) = 0.00, *p* = .999, *Δ*CFI = 0.001, *Δ*RMSEA = 0.001, *Δ*SRMR = 0.000). Thus, we concluded that our scale displayed similar psychometric properties across both measurement occasions.

### Discussion

Study 2 yielded several noteworthy findings. First, our structural analyses revealed that a four-factor model best fitted the data at hand. These factors were remarkably similar to those of the original JLS scale–with the exception of the *negative attitudes towards/negative evaluation of the outgroup* factor, which is broader and more outgroup-directed than the original *negative views towards intergroup relations factor*. Secondly, correlational analyses provided support for our final scale’s nomological validity, as it was shown to relate to various facets of Black African identity, intergroup contact, friendships and attitudes and societal views in the hypothesized directions. Thirdly, measurement invariance testing provided evidence for the temporal stability of the AWB scale–thereby highlighting its test-retest reliability.

Taken together, Studies 1 and 2 provided converging evidence for the factor structure of anti-White bias in the UK and the structural qualities of our new measure. Moreover, the Study 2 results additionally demonstrated its convergent and discriminant validity. Having established these psychometric properties of our scale, in Study 3, we moved on and examined the relationships between AWB and reactions to race-related contemporary events. Hence, we included a variety of topics that either reflected actual societal events (e.g., the destruction of colonial statues) or were inspired by actual societal events (e.g., a fictional report of a White policeman shooting a Black male; which was adapted from Cooley et al. [72]). The main inclusion criteria for these topics were that they (1) had a salient ‘racial’ component, (2) left some aspects open for interpretation–thereby allowing for some variability in biased responding–, and (3) could theoretically be expected to touch upon one or multiple facets of anti-White bias. For each reaction to a specific topic, we formulated our predictions concerning the magnitude of the relationship with AWB. These were based on the findings of Johnson and colleagues [20, 35]. For example, because these scholars had observed relations between JLS and perceived anti-Black discrimination ranging from .33 to .40, we predicted *r* = .30 (or larger) for all items reflecting attributions of negative outcomes for Black people to discrimination.

## Study 3

### Method

#### Participants

Study 3 was preregistered at https://aspredicted.org/blind.php?x=ja9vf5. Power calculations were based on the smallest preregistered predicted correlation, i.e., between AWB and reactions to societal events on the other (*r* = .15; see “Measures”). Our analyses revealed that a sample of *N* = 346 would suffice to achieve 80% power to detect an effect of a similar magnitude. Anticipating some dropout, we oversampled and recruited 423 Black UK participants on Prolific (2£ compensation), of which 300 (71.0%) completed both parts of our study (see below). We dropped *n* = 59 because they did not fulfill at least one of our inclusion criteria (which were analogous to Studies 1 and 2). A further *N* = 40 participants were excluded because they either failed at least one out of four attention checks (e.g., “Please select response [X] for this question”), or at least one manipulation check (see ‘Procedure’). Our final sample thus consisted of *N* = 201 participants (75 males/123 females/3 non-binary; age: *M* = 29.3, *SD* = 9.26, range = 18–57). A sensitivity analysis revealed that this sample size achieved 80% to detect effects of size *r* = .20.

The majority of our sample had obtained a Bachelor’s (51.2%) or high school degree (28.4%; Master’s degree: 17.4%; no degree: 1.0%; PhD: 2.0); and most participants reported a monthly salary of >2,500€ (72.6%). Furthermore, on average participants rated themselves as 3.18 (*SD* = 1.33) on a 7-point self-placement scale of political ideology (1 = left, 7 = right).

#### Procedure

Participants were solicited for a two-part survey about “decision-making processes”. In the first part of the questionnaire, they completed our AWB scale, and they provided some demographic information. For exploratory purposes, our participants also completed the HEXACO personality inventory (for all analyses involving this variable, we refer the reader to the Supplementary Online Materials on our Open Science Webpage). Within a week, they were contacted again and we kindly invited them to complete our new survey. To decrease the attrition rate, three reminders were sent out. Data collection was terminated three days after the last reminder had been sent out. This second survey consisted of three parts.

First, participants read a police report about a White police officer shooting a Black man, which was adapted from Cooley et al. [72]. Importantly, this report entailed a rather large degree of ambiguity with respect to whether or not the police officer had a reasonable cause to be suspicious and to act in the way described. We reasoned that leaving some details to the imagination of the reader would maximize the potential of the manipulation to distinguish between those low and high in anti-White bias. Subsequently, participants completed some items assessing their reactions to the depicted event.

Secondly, participants were presented a news article describing the real case of a Belgian politician, who allegedly had committed large-scale fraud with government subsidies. Critically, it was conveyed that the suspect was of North African descent The main reason we included this scenario was to assess whether our AWB scale would predict the attribution of a negative outcome for a “peripheral” group member (i.e., someone who does not belong to the same racial group as the participants, but who is a minority group member, just like them, nonetheless) to discrimination to a similar extent as JLS [20, 35] predicted attribution of a negative outcome for a fellow Black person to discrimination. Moreover, we also informed our participants that the money had been requested to fund a non-profit organization, owned by the politician, to take minority youth off the streets and involve them in various social projects. It was assumed that by highlighting this race-related content we would “activate” biased responding among those high in anti-White bias. As in the first scenario, we left a degree of ambiguity by not explicitly answering the question of whether the suspect was actually guilty (also in reality, no legal decision has yet been taken). As before, participants then completed some items assessing their reactions to the depicted event.

In the final part of the survey, participants were confronted with some actual contemporary societal events and topics. These were (1) the 2021 action plan of the EU to combat structural discrimination, (2) the 2010 statement by German chancellor Angela Merkel about the “bankruptcy” of multicultural society, (3) a report describing the increasing employment gap between majority and minority group members in Europe, (4) recent destructions of statues of the colonial-era monarch Leopold II, who ruled and exploited the colony of what is now known as the Democratic Republic of Congo, (5) the 2021 social media boycott by the Premier League to combat structural discrimination in soccer, (6) the European migration situation, and (7) affirmative action targeting Black people. Importantly, these events all alluded to either (structural) discrimination, addressing (structural) racism and/or race-related topics, and hence, reactions to these events could thus reasonably be expected to relate to one or multiple facets of our bias measure as well. At the end of the survey, participants were asked whether they could guess the study’s hypothesis. Subsequently, they were debriefed and thanked for their cooperation.

#### Measures

Unless stated differently, items were responded to on five-point Likert scales, ranging from 1 = “completely disagree” to 5 = “completely agree”.

Participants completed the same 23-item **AWB** scale as in Studies 2 and 3 (*M* = 2.71, *SD* = 0.70; *α* = .93). For the **police shooting report**, three items assessed the extent to which participants *attributed* the behavior of the White police officer *to discrimination* (e.g., “The suspect would not have been shot if he were a different race”; *M* = 3.77, *SD* = 1.19, *α* = .91). Another two items were implemented to measure *approval of [ambiguously] racist behavior* (e.g., “I support the actions of the police officer”; *M* = 2.13, *SD* = 1.12, *r* = .83). *Perpetrator-directed punitive responding* and *perceived perpetrator responsibility* were measured with four (e.g., “The police officer should be criminally indicted”; *M* = 3.37, *SD* = 1.14, *α* = .93) and three items, respectively (e.g., “I feel that the police officer is fully responsible for the injuries to the suspect”; *M* = 3.75, *SD* = 0.89, *α* = .73), and *emotions towards the perpetrator* (positive emotions: 3 items [e.g., “sympathy”]; *M* = 1.85, *SD* = 0.94, *α* = .92; negative emotions: 3 items [e.g., “anger”]; *M* = 3.31, *SD* = 1.25, *α* = .93) and *the victim* (positive emotions: *M* = 3.14, *SD* = 1.08, *α* = .92; negative emotions: *M* = 1.93, *SD* = 0.89, *α* = .86) were also gauged.

For the **fraud scenario, t**hree items assessed the extent to which participants *attributed* the allegations *to discrimination* (e.g., “This person would not have been accused if she were a different race/ethnicity”; *M* = 3.04, *SD* = 1.00, *α* = .83). A single item measured *justification of the behavior* (“I support the actions of this person”; *M* = 2.69, *SD* = 1.02). *Perpetrator-directed punitive responding* and *perceived perpetrator responsibility* were measured with four (e.g., “This person should be criminally indicted”; *M* = 2.91, *SD* = 0.88, *α* = .90) and three items respectively (e.g., “I feel that this person is fully responsible for her actions and should be charged with fraud”; *M* = 2.85, *SD* = 0.77, *α* = .70), and *emotions towards the perpetrator* (positive emotions: *M* = 2.77, *SD* = 1.04, *α* = .93; negative emotions: *M* = 2.31, *SD* = 0.93, *α* = .88) were also gauged.

With respect to **societal events**, Table 8 provides an overview of sample items for all measured reactions to these events, together with means, *SD*s and reliabilities. We also preregistered our expectations with respect to the relationships between AWB and the reactions to societal events. Our predictions were based on the relationships obtained in studies validating the JLS [20, 35]. For example, these authors found that the JLS significantly predicted perceived racism in ambiguous situations (minimum *β* = .30). Hence, we anticipated a relationship of at least .30 between AWB and all sets of items reflecting attributions to discrimination–see Table 8, second last column, for a full overview of preregistered correlations.

**Table 8:** Thematic overview of reactions to societal events (Study 3), sample items, and preregistered and observed correlations with AWB, per topic.

Event/Topic	Reaction (number of items) + Sample item	*M*	*SD*	*α/r*	Preregistered	Observed
					*r*	*r*
1. Action plan of the EU to combat structural discrimination						
	1. Need for such measures (4)	4.51	0.94	.77	.30	.24***
	*“Such an action plan is much needed”*					
	2. Doubting the usefulness (2)	3.26	1.00	.84	.30	.14
	*“I do not think that the action plan will make a big difference”*					
	3. Doubting the intentions (1)	3.19	1.08	*na*	.30	.18**
	*“I believe that the action plan by the EU to combat structural racism is "window dressing”*					
2. Statement by German chancellor Angela Merkel about the “bankruptcy” of the multicultural society						
	1. Attributions to discrimination (2)	3.06	0.76	-.11	.30	.21**
	*“If this statement were to be true*, *it would be because of EU majority citizens opposing the multicultural society”*					
	2. Agreement with statement (3)	3.42	0.91	.78	.00	.15*
	*“I agree with this statement”*					
3. Report describing the increasing employment gap between European majority and minority group members						
	1. Attributions to discrimination (3)	4.01	0.90	.88	.30	.45***
	*“The employment gap described in the report reflects structural racism”*					
	2. Attributions to other factors (2)	3.01	1.15	.88	-.30	-.06
	*“The employment gap described in the report reflects differential educational attainment between the majority and ethnic minority populations in the EU”*					
4. Destructions of statues of the colonial-era monarch Leopold II						
	1. Punitive responding towards protesters (2)	2.67	1.29	.77	-.30	-.39***
	*“Those who sprayed the graffiti should be criminally indicted”*					
	2. Sympathy for protesters (2)	3.82	1.20	.81	.30	.30***
	*“I can understand the anger of those who sprayed the graffiti”*					
	3. Punitive responding towards Belgium (3)	3.88	1.04	.80	.30	.40***
	*“Belgium should financially compensate Congo for what happened during the colonization of Congo”*					
	4. Support for cause protesters (3)	3.29	1.05	.68	30	.46***
	*“I support the cause of those who sprayed the graffiti*.*”*					
5. Social media boycott by the Premier League to combat structural discrimination in soccer						
	1. Need for such measures (3)	3.52	1.04	.85	.30	.05
	*“Such a social media boycott is much needed”*					
	2. Doubting the usefulness (2)	3.80	1.06	.85	.30	.18**
	*“I do not think that the social media boycott will make a big difference”*					
	3. Doubting the intentions (1)	3.65	1.19	*na*	.30	.10
	*“I believe that the action plan by the EU to combat structural racism is "window dressing”*					
6.European migration “crisis”						
	1. Attitudes towards stricter policies (general immigration; 3)	2.71	0.77	.64	.00	-.11
	*“Should the EU go through an economically difficult period*, *it is imperative that the number of new immigrants should be limited*.*”*					
	2. Attitudes towards stricter policies (African immigration; 3)	2.54	0.80	.66	-.30	-.22**
	*“Should the EU go through an economically difficult period*, *it is imperative that the number of new immigrants from African countries should be limited*.*”*					
7. Affirmative action targeting Black people						
	1. Importance of such measures (10)	3.35	0.85	.90	.30	.38***
	*“Companies should be obliged to employ a minimum number (“quotas”) of Black minority members*.*”*					

*Notes*. Cronbach’s Alpha is reported for measures with >2 items (else coefficient refers to *r*).

*: *p* < .05.

**: *p* < .01.

***: *p* < .001.

### Data-analysis and results

#### Police shooting report

Correlational analyses revealed that AWB was positively related to attributions of the officer’s behavior to discrimination (*r* = .46, *p* < .001), perceived responsibility (*r* = .33, *p* < .001) and punitive responding (*r* = .37, *p* < .001), and AWB related negatively to justification of the officer’s behavior (*r* = -.31, *p* < .001). Moreover, AWB was also related to emotions towards the shooter (positive emotions: *r* = -.32, *p* < .001; negative emotions: *r* = .34, *p* < .001) and the suspect (positive emotions: *r* = .22, *p* = .002).

#### Fraud news article

Correlational analyses revealed that AWB was related to attributions of the allegations to discrimination (*r* = .32, *p* < .001). No other relationships were found to be significant (all *p*s > .175).

#### Reactions to other societal events

**Fig 1:**
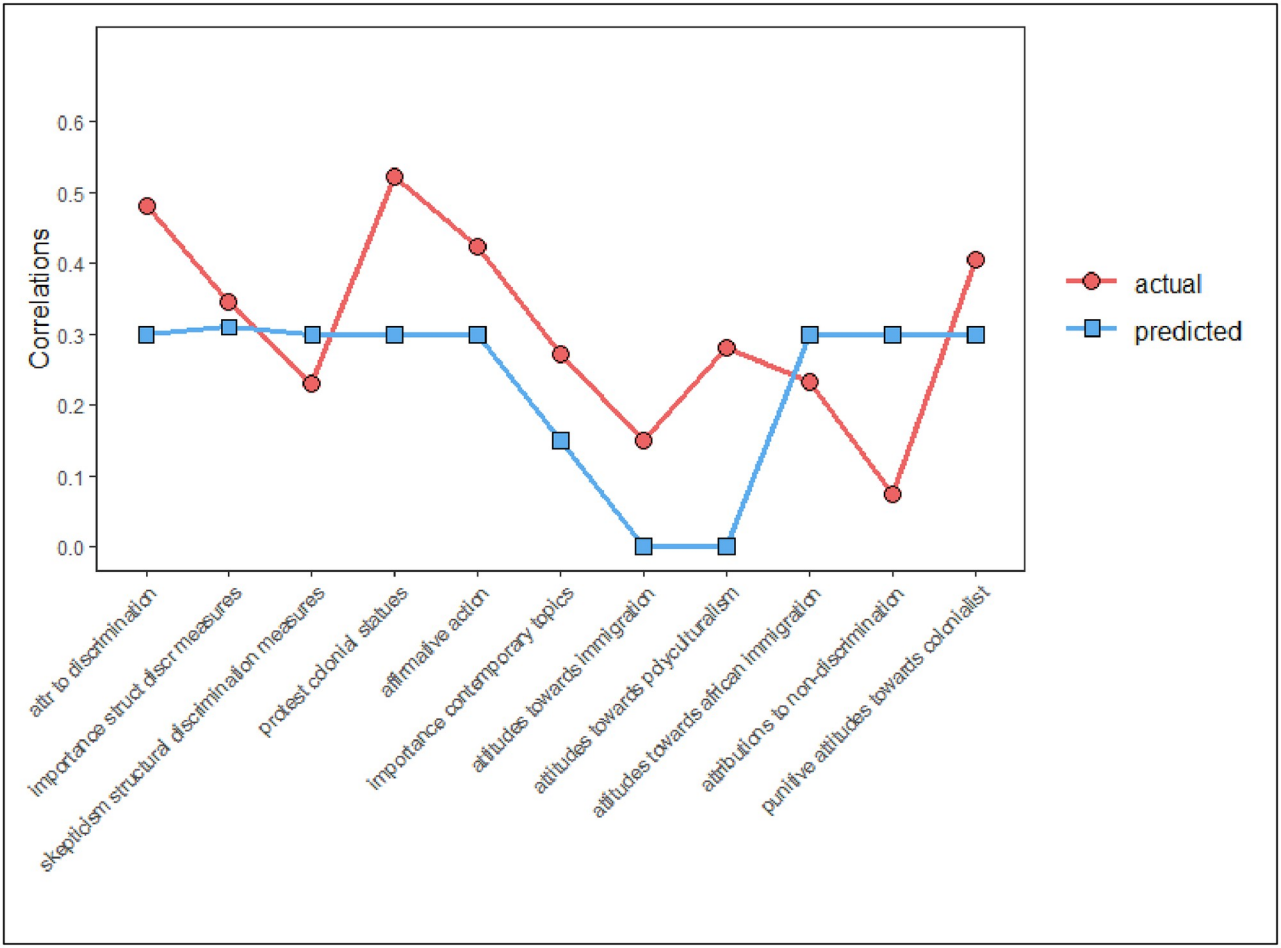
Comparison of actual (red line) and observed (blue line) canonical correlations between AWB and sets of items sharing an underlying theme.

Table 8 (last column) provides an overview of the simple correlations between anti-AWB and the various reactions to societal events. To summarize the pattern of relationships, we additionally computed canonical correlations between our scale and sets of items reflecting the same underlying theme. For example, in the set *attributions to discrimination* we included both items attributing the “failure” of the multicultural society to discriminatory acts by majority citizens, and items attributing the European employment gap (between majority and minority group members) to widespread discriminatory practices. As such, 11 sets of items with a similar underlying theme were identified (see below). A canonical correlation was computed for each set, using the CCA package v1.2.1 [73] and CCP package v1.1 [74] in R. The results of this analysis are graphically depicted in Fig 1, and our predicted correlations are added as a point of reference.

Results revealed that AWB significantly predicted all sets of outcome variables (*r*s ranging from .23-.52, all Wilks’ *λ*s < 0.96, all *p*s < .026), with the exceptions of *the importance of contemporary topics* (*r* = .15, Wilks’ *λ*s = 0.98, *p* = .141) and *attribution to other factors than discrimination* (*r* = .07, Wilks’ *λ*s = 0.99, *p* = .516). Moreover, the magnitude of the canonical correlations exceeded our expectations for most outcomes, with the noteworthy exceptions of *skepticism about measures to tackle structural discrimination* (predicted = .30, observed = .23), *attitudes towards immigration from African countries* (predicted = .30, observed = .23) and *attributions to other factors than discrimination* (predicted = .30, observed = .07).

### Discussion

The results of Study 3 were mainly in line with our preregistered hypotheses. Specifically, AWB was significantly related to reactions to a police shooting (of an unarmed Black man) report, to fraud allegations towards a minority group member, and various relevant societal events. Interestingly, it was found that most of the observed relations were at least as large, or larger than those we had anticipated based on prior research. As such, the Study 3 results thus provided converging evidence for the predictive validity of our AWB scale for reactions to societal issues.

## General discussion

The central goal of the present study was to construct and validate an instrument to examine anti-White bias among Black minority group members living in the UK. To enable the study of intergroup bias in the UK context, we adapted the Johnson & Lecci questionnaire for anti-White bias [20] and validated the new AWB scale among Black UK citizens. Whereas Studies 1 and 2 demonstrated our scale’s adequate psychometric properties, Study 2 also provided converging evidence for its temporal stability, and for its construct validity by examining the relationships with variables closely related to the concept. Subsequently, in Study 3, anti-White bias was shown to be related to various self-reported reactions to race-related contemporary societal events. Taken together, our results thus demonstrate (1) the applicability of our AWB measure, and (2) the utility of anti-White bias to explain and understand reactions vis-à-vis relevant race-related societal events.

The present results make various theoretical contributions to literature on intergroup bias. In what follows, we will first summarize findings that speak to the universality of anti-White bias. Then, we will recapitulate those results that are relevant to the comparison with anti-minority bias from the viewpoint of the majority.

### The universality of anti-White bias

Outgroup bias is a complex phenomenon determined by a multitude of factors [75, 76] such as migration background [30] and historical intergroup tensions with the (White) majority [77]. Given that the Black UK population differs substantially in terms of such factors from the Black American community under study by Johnson and colleagues [20, 35], we did not a priori expect our findings to mirror the observations made by these scholars. Yet, despite these considerable inter-minority-group differences, there appear to be substantial similarities. For example, the Study 1 results provided some preliminary evidence that the constellation of anti-White biased attitudes among Black UK people resembles that of anti-White biased attitudes among Black American people, with three out of the four facets of the original JLS emerging from our ESEM analyses. Furthermore, in Study 3, we obtained similar relationships between anti-White bias and reactions to specific race-related events–targeted to the European context–as Johnson and Lecci [20] and Johnson and colleagues [35], who investigated hypothetical scenarios. Interestingly, the observed pattern of canonical correlations was remarkably similar to what we predicted, with most relationships achieving the a priori hypothesized size and direction. The latter finding thus indicates that anti-White bias relates to reactions to race-related events to a similar extent in the UK versus the American societal setting.

In conclusion, our results suggest that the experience of anti-White bias is remarkably similar in the UK context, compared to the U.S societal landscape. As such, our results seem to testify to the universality of the experience of being a Black minority group member in a predominantly White society. In the limitations and directions for future research section below, we return to this issue and explain how the cross-cultural comparability of minority experiences can be further investigated.

### Similarities and differences with majority member biases

Our results further call for a comparison of the biases held by majority and minority group members. A first most noteworthy observation is that anti-White bias seems to consist of (at least) four distinguishable facets, some of which are predominantly outgroup-directed (e.g., negative attitudes and evaluations of the outgroup) and others which are both outgroup-driven and ingroup-directed (e.g., perceptions of White anti-Black racism). On the one hand, the complexity of these racial attitudes starkly contrasts with the negative intergroup views of majority group members, which consistently comprise a single dimension [33] or two dimensions [78]. On the other hand, this multidimensionality aligns well with the scholarly reflections and empirical observations of Brigham [34] and Johnson and Lecci [20].

Secondly, some similarities with majority member biases also emerged. For example, the Study 2 results revealed that anti-White bias among Black minority group members relates to various social categorization and identification variables, quality and quantity of intergroup contact, and discrimination and unfairness perceptions, all of which have previously been associated with anti-minority bias among White people. For example, both outgroup homogeneity [79] and essentialist entativity [65] have been shown to strongly predict anti-minority bias. In a similar vein, quality and quantity of contact with minority members have frequently been associated with reduced prejudice towards minority groups [62], a finding that materialized in our Black samples as well. Finally, Study 4 also revealed that some of the reactions to race-related events of those high in anti-White bias mirrored responses of anti-minority prejudiced individuals to similar topics. For example, it was found that anti-White bias was positively associated with both skepticism about multicultural societies and attempts to promote living together in diversity. This finding is analogous to the criticisms towards multiculturalism and multicultural policies expressed by those high in racial minority-directed prejudice [80, 81]. Relatedly, the obtained positive relationship between outgroup perpetrator punitive responding and anti-White bias (police shooting) and the negative relationship between ingroup perpetrator sympathy and anti-White bias (fraud article) respectively mimic the inflated outgroup hate and ingroup favoritism observed among racially prejudiced majority members [58]. Taken together, these results additionally suggest the generalizability of biases and their correlates across minority and majority cultures.

### Limitations and directions for future research

The present study also suffers from some limitations. First, although the cultural generalizability, validity and predictive utility of our anti-White bias scale was extensively assessed across three different samples, it should be noted that our measure did not contain any reverse-keyed items. Absence of items expressing lack of bias may lead to contamination of a scale by acquiescence variance, which can inflate inter-item correlations and the relations with other scales having the same lack of reverse-keyed items.

Secondly, the focus of the present study did not extend to other racial-ethnic minorities than Black UK citizens. In fact, we are aware of only one study [19] that explicitly pitted the biases of two minority groups–European Black people and European Muslim people–against each other. Interestingly, these scholars also revealed some similarities between both groups in mean anti-White prejudice ratings and the relationship with trait emotional intelligence–thereby corroborating our reflections on the premise of cross-minority generalizability of biased attitudes. In any case, the literature on intergroup negativity would undoubtedly benefit from research programs exploring biases held by a variety of other racial, ethnic, and cultural minorities, to evaluate their similarities and differences. Relatedly, we acknowledge that the present study only explored one specific type of bias–i.e., towards White majority group members–and that we did not lay our scope on *inter-minority* biases. Yet, it can be argued that such biases deserve scholarly attention in their own right, because they are highly relevant to intergroup relations in increasingly diverse societies as well. Moreover, such research endeavors could result in a more fine-grained theoretical understanding of the cultural specificity and generalizability of intergroup negativity too, by revealing some commonalities as well as differences with anti-White bias. For example, whereas anti-White bias may be fueled by genuine concerns such as widespread structural discrimination of disadvantaged groups, inter-minority bias may emanate from other (genuine or symbolic) apprehensions and may therefore have a different constellation. In order to facilitate such comparisons across various types of bias, we strongly encourage future researchers to investigate minority group members’ biased attitudes against their minority group counterparts.

Thirdly, it should be acknowledged that, in the current study, we only sampled Prolific participants. Although the Prolific crowdsourcing platform has repeatedly been shown to be a highly reliable source of data [82, 83], relying on survey panel-based samples–all pertaining to the same opt-in panel–can also be tedious and may obscure trends present in the general population. Future research could undoubtedly benefit from the inclusion of other types of data (e.g., student samples, nationally representative panels) to study the biases held by minority group members.

## Conclusions

Despite the considerable increase in racial-ethnic diversity in Western societies over recent decades, minimal empirical focus has been given to the experiences and attitudes of minority group members. The present study tackled this lacuna in the literature by developing a new measure to study anti-White bias. and by investigating the role played by biased attitudes in the development of reactions to contemporary race-related societal events. Our findings uncovered some similarities between the biases held by American and UK Black people and some common grounds. At the same time, our results also revealed some relevant distinctions. Taken together, the present results thus support both accounts of cross-cultural generalizability as well as cultural specificity of intergroup bias.
