# Correction: Adaptation and validation of the Johnson-Lecci scale to assess anti-white bias among black UK minority group members

**DOI:** 10.1371/journal.pone.0305184

**Published:** 2024-06-04

**Authors:** Kim Dierckx, Alain Van Hiel, James D. Johnson, Len Lecci, Barbara Valcke, Eva Kefilwe Sekwena

The affiliation for the third author is incorrect. The correct affiliation is not indicated. James D. Johnson is not affiliated with #2 but with: The Weber Group, 4 Starling Street, Burleigh Heads, Queensland 4220, Australia (ABN 28632112843).
